# A fast analysis method for non-invasive imaging of blood flow in individual cerebral arteries using vessel-encoded arterial spin labelling angiography

**DOI:** 10.1016/j.media.2011.12.004

**Published:** 2012-05

**Authors:** Michael A. Chappell, Thomas W. Okell, Stephen J. Payne, Peter Jezzard, Mark W. Woolrich

**Affiliations:** a Institute of Biomedical Engineering, University of Oxford, Old Road Campus, Headington, Oxford OX3 7DQ, UK; b FMRIB Centre, Nuffield Department of Clinical Neurosciences, University of Oxford, John Radcliffe Hospital, Headington, Oxford OX3 9DU, UK

**Keywords:** Arterial spin labelling, Dynamic angiography, MRI, Vessel selective

## Abstract

Arterial spin labelling (ASL) MRI offers a non-invasive means to create blood-borne contrast *in vivo* for dynamic angiographic imaging. By spatial modulation of the ASL process it is possible to uniquely label individual arteries over a series of measurements, allowing each to be separately identified in the resulting angiographic images. This separation requires appropriate analysis for which a general Bayesian framework has previously been proposed. Here this framework is adapted for clinical dynamic angiographic imaging. This specifically addresses the issues of computational speed of the algorithm and the robustness required to deal with real patient data. An algorithm is proposed that can incorporate planning information about the arteries being imaged whilst adapting for subsequent patient movement. A fast maximum *a posteriori* solution is adopted and shown to be only marginally less accurate than Monte Carlo sampling under simulation. The final algorithm is demonstrated on *in vivo* data with analysis on a time scale of the order of 10 min, from both a healthy control and a patient with a vertebro-basilar occlusion.

## Graphical abstract

**Graphical abstract:**

**Figure:**
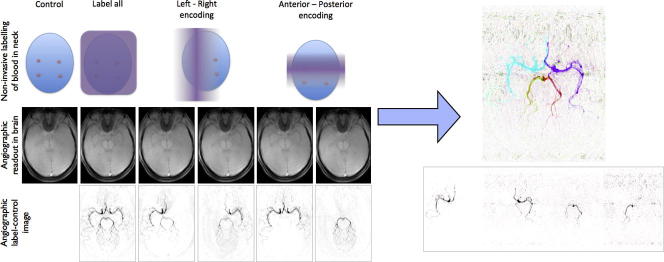

**Highlights:**

► Non-invasive vessel selective MR angiography. ► Fast Bayesian analysis for artery flow contributions. ► Robust treatment of imperfect artery location specification e.g. due to patient movement. ► Robust to clinical scenarios, e.g. occluded arteries.

## 1 Introduction

Arterial spin labelling (ASL) magnetic resonance imaging is an entirely non-invasive means to measure blood flow in the body. In ASL an endogenous ‘contrast agent’ is generated by radio-frequency inversion of the magnetization of flowing blood upstream from the organ being investigated, followed by subsequent imaging of this labelled blood once it has reached the organ. This image is subtracted from another taken in the absence of labelling to remove the static tissue signal and reveal the blood supply, from which quantitative measures of blood flow can be derived (Alsop and Detre, 1996; Buxton et al., 1998). The ASL contrast mechanism can also be applied to angiographic imaging of the vessels, including dynamic (‘cine’) acquisitions (Edelman et al., 1994; Wang et al., 1991).

**Fig. 1:**
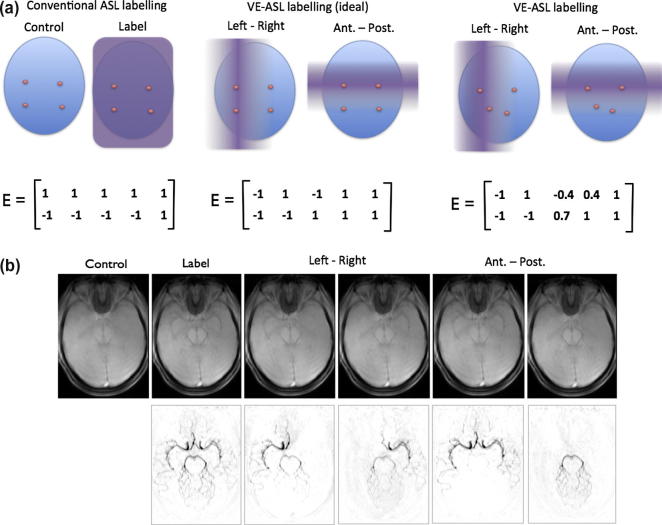
A guide to VE-ASL dynamic angiography. (a) Labelling in the neck and the associated encoding matrix for four arteries (right and left internal carotids, right and left vertebrals) plus static tissue contribution. In conventional ASL all the arteries are either in the control or labelled condition. In VE-ASL the labelling is modulated to put a subset in control whilst the others are labelled. Ideally arteries will be either fully labelled or controlled, but in practice it may not be possible to achieve this and in some acquisitions arteries will be partially labelled. (b) Example VE-ASL angiographic images in the brain showing raw image (dominated by static tissue contribution) and result after subtraction from the control image.

Some organs, most notably the brain, are supplied with blood by a number of arteries, each artery supplying a different region; commonly referred to as vascular territories. It is of clinical relevance to be able to visualise the blood flow within individual arteries and the territories they supply. A notable example is collateral flow in the ‘Circle of Willis’ whereby blood may pass from one major artery via a communicating artery to feed a different vascular territory. For example, flow from an internal carotid artery may be diverted to a posterior territory normally supplied by the vertebral arteries in the case of vertebral occlusion or stenosis. A number of selective ASL labelling methods have been proposed that can target individual arteries (Helle et al., 2010a, 2010b; Dai et al., 2010; Davies and Jezzard, 2003; Zimine et al., 2006; Hendrikse et al., 2004). Recently a more efficient strategy has been demonstrated for the simultaneous labelling of multiple arteries (Günther, 2006; Wong, 2007), termed vessel-encoded ASL (VE-ASL). This method can be used to produce vessel-selective dynamic angiograms of the major cerebral arteries (Okell et al., 2010). An example of VE-ASL labelling is given in Fig. 1. Conventional ASL fully inverts the blood in all arteries within the labelling regions. Subtraction of the subsequent image from a control, in the absence of labelling, produces an image of flowing blood. VE-ASL spatially modulates the inversion process such that in one acquisition within a subset of arteries the blood will be inverted and the remainder will remain in the unlabelled (control) condition. Over a number of such acquisitions, with different modulations, each artery will have been uniquely encoded and its contribution to the blood flow image can be extracted in post-processing. The most straightforward approach to post-processing such data involves simple addition or subtraction of images (typically written as the equivalent matrix operation). However, more complex encodings and imperfections in the modulation, for example due to non-ideal locations of the arteries within the labelling region, necessitate more careful analysis (Chappell et al., 2010; Wong, 2007). This typically involves the specification of the mixing or encoding matrix (Fig. 1) for the different artery contributions under the encoding cycles employed in the acquisition. The (Moore–Penrose) pseudo-inverse of this matrix can then be used in post-processing to calculate individual artery contributions to the flow.

We have previously proposed a general framework for the analysis of VE-ASL data employing a Bayesian solution (Chappell et al., 2010). It offered a number of advantages over existing matrix inversion approaches. In particular, it provided a full model for the relationship between the locations of the arteries in the labelling plane and the encoding profile, allowing the locations to be inferred from the data whilst permitting prior information from planning acquisitions to be incorporated. Furthermore, a voxel-wise classification with *N*-arteries per class was used to restrict the number of arteries that were assumed to contribute to the signal in a voxel, resulting in greater signal-to-noise ratio (SNR) efficiency and reductions in the number of encoding cycles required.

The general framework was demonstrated on VE-ASL imaging of the cerebral vascular territories (Chappell et al., 2010). However, a number of limitations were present in the existing framework that become more acute in the case of angiographic data, particularly when this is coupled with clinical application. Firstly, the original proposal employs a Markov Chain Monte Carlo (MCMC) sampling procedure to infer the ‘global’ parameters, e.g. the artery locations in the labelling region. This results in a relatively long computation time, something that will be more pronounced in higher resolution angiographic data. Secondly, while there are potential benefits in fully inferring the artery locations from the data, in a clinical setting it may be more beneficial to constrain the artery locations based on information obtained in the planning phase and according to variation that might be expected due to patient movement. For example, constraining the artery locations by only permitting a global three degree-of-freedom transformation. This will result in fewer parameters in the analysis and so should be more robust to the poorer SNR and greater frequency of motion artefacts expected of clinical data, which is caused by greater decay of the ASL label during the delayed blood transit, typical of patients with cerebrovascular disease. Additionally, in patients with highly stenosed arteries that provide little signal downstream, allowing completely free determination of the artery locations leaves the analysis vulnerable to bias by artefacts and incorrect assignment of the signal components. In these cases it may sometimes be critical to differentiate between low flow and zero flow, so a method that is robust to small signals from some arteries is desirable.

In this work we present a number of modifications to the existing general framework for VE-ASL analysis that address the issues arising from its use in dynamic angiographic imaging. Firstly, we employ a Maximum *A Posteriori* (MAP) solution for the ‘global’ parameters to address issues of computation time. Secondly, we introduce constraints into the inference of artery locations that match those that might be expected from subject movement. Finally, we offer a solution to the general framework that does not involve the computationally costly solution of the global parameters, yet retains many of the robust characteristics of the general framework. This latter option offers a routine alternative to matrix inversion analyses with very minimal increase in computational cost.

## 2 Theory

The full theory for VE-ASL analysis is given in (Chappell et al., 2010), but a summary of the key ideas and equations is given here.

### 2.1 Encoding matrix representation

The tag-control differencing of VE-ASL can be represented in matrix form (Wong, 2007):

(1) s=Ef

where **f** represents the sources of signal, **s** is the vector of (noise-less) measured values and **E** is the encoding matrix:

(2) $$E=\left[\begin{array}{cccc} m_{11} & \cdots & m_{1M} & 1\\ \vdots & \ddots & \vdots & \vdots \\ m_{N1} & \cdots & m_{\mathrm{NM}} & 1 \end{array}\right]$$

where *N* encoding cycles produce *N* images by spatially encoding the signal from *M* arteries. A conventional control-all image in cycle *i* would be generated by setting *m_ij_ *= 1 for each vessel, *j*, and likewise for a tag-all image by setting *m_ij_* = −1. The final column must always be all unity values, since this dictates the contribution of the static magnetization of the tissues to the measured signal. Example encoding matrices for labelling arteries in the neck are shown in Fig. 1a.

### 2.2 *N*-artery classification

The full encoding matrix permits any voxel to receive contribution from all supply arteries. However, this is unlikely in practice since most tissue will be fed by only a subset of the labelled arteries. Analysis should ideally be performed only with a subsection of the encoding matrix representing the appropriate arteries, leading to a better-conditioned encoding matrix and more robust analysis. This can be achieved by including classification within the analysis, where each voxel is assigned a class, each class specifying a unique subset of the supply arteries. This can be described for the *c*th class using a matrix, *P_c_*, in the *k*th voxel:

(3) $$s_{k\text{,}c}={\mathrm{EP}}_{c}f_{k\text{,}c}$$

If we are considering *L_c_* arteries in the *c*th class, then *f_kc_* will be (*L_c_ *+ 1) × 1 in size. *P_c_* will be a matrix of zeros, (*M *+ 1) × (*L_c_ *+ 1) in size, with a single unity entry in each column that selects individual vessels included in this class (plus the static magnetization in the final column). For example, for a 3-artery problem (*M = *3), considering pairs of arteries (*L*_c_ = 2) the *P_c_* matrices for all classes (combination of arteries) are:

(4) $$P_{c}=\left\{\left[\begin{array}{ccc} 1 & 0 & 0\\ 0 & 1 & 0\\ 0 & 0 & 0\\ 0 & 0 & 1 \end{array}\right]\text{,}\left[\begin{array}{ccc} 1 & 0 & 0\\ 0 & 0 & 0\\ 0 & 1 & 0\\ 0 & 0 & 1 \end{array}\right]\text{,}\left[\begin{array}{ccc} 0 & 0 & 0\\ 1 & 0 & 0\\ 0 & 1 & 0\\ 0 & 0 & 1 \end{array}\right]\right\}\text{,}$$

In essence the *P* matrices mean that only a section of the encoding matrix is considered at a time.

### 2.3 Bayesian framework

The estimation of voxelwise class membership and flow contributions can be cast within a Bayesian framework. Assuming white noise the Likelihood, as in (Chappell et al., 2010), is given by:

(5) $$\mathrm{Pr}(T|F\text{,}E\text{,}q=\kappa \text{,}\phi )=\underset{k}{\prod }\mathrm{Pr}(t_{k}|f{\;}_{k}\text{,}E\text{,}q_{k}=\kappa_{k}\text{,}\phi_{k})\text{,}$$

(6) $$\mathrm{Pr}(t_{k}|F_{k}\text{,}E\text{,}q_{k}-\kappa_{k}\text{,}\phi_{k})=\frac{\phi_{k}^{N/2}}{{\left(2\pi \right)}^{N/2}}e^{-\frac{\phi_{k}}{2}{\left(t_{k}-s_{k\text{,}\kappa_{k}}\right)}^{T}(t_{k}-s_{k\text{,}\kappa_{k}})}\text{,}$$

(7) $$t_{k}=s_{k\text{,}\kappa_{k}}(t)+e_{k}(t)$$

(8) $$e_{k}(t)∼\prod N(0\text{,}\phi_{k}^{-1})$$

where *k* refers to an individual voxel, ***t**_k_* is the measured data containing *N* measurements, **q** is the map of discrete class labels and **κ** is a specific configuration thereof, thus *κ_k_* is a specific class label assigned to voxel *k*, ***ϕ*** is the vector of noise precisions.

Application of Bayes’ theorem gives the posterior distribution:

(9) Pr(F,E,q=κ,ϕ|T)∝Pr(T|F,E,q=κ,ϕ)Pr(F,ϕ)Pr(E)Pr(q=κ).

with the following priors:

(10) $$\begin{array}{cc} & \mathrm{Pr}(F\text{,}\phi )=\phi^{\frac{L_{c}}{2}}|P_{c}^{T}E^{T}{\mathrm{EP}}_{c}^{T}{|}^{1/2}\\ & \mathrm{Pr}(q_{k}=c|\pi_{c})\pi_{c}\\ & \sum \pi_{c}=1\\ & \mathrm{Pr}(\pi_{c})=1/|\pi_{c}| \end{array}$$

The prior on the precisions and flow estimates is the joint Jeffrey’s prior for both (Lee, 1997), making the prior uninformative for these parameters. The prior probability for a voxel belonging to class *c* is set to be the proportion of voxels belonging to that class, *π_c_*, as determined from the data. The prior specification includes the requirement that the class proportions must sum to unity. Additionally an automatic relevancy determination (ARD) prior is placed on each class proportion so that where data does not support that class it will be automatically removed from the model (Mackay, 1995). Finally a prior needs to be set for the encoding matrix; ideally the full encoding matrix is defined *a priori* by the acquisition and from the planning angiographic image at the labelling region (see below), in which case Pr(**E**) = 1. The encoding matrix may be further parameterised, for example in terms of the locations of the arteries in the labelling plane. The encoding matrix parameters being inferred from the data and subject to prior information defined within the Pr(**E**) term.

Marginalization can be performed analytically over all the parameters that vary from voxel to voxel: flow, noise precision and class label in every voxel. This gives:

(11) $$\mathrm{Pr}(E\text{,}\pi |Y)\propto \underset{k}{\prod }\left[\underset{c}{\sum }\left\{\pi_{c}[{\left(y_{k}-{\mathrm{EP}}_{c}{\left(P_{c}^{T}E^{T}{\mathrm{EP}}_{c}\right)}^{-1}P_{c}^{T}E^{T}y_{k}\right)}^{T}{\left(y_{k}-{\mathrm{EP}}_{c}{\left(P_{c}^{T}E^{T}{\mathrm{EP}}_{c}\right)}^{-1}P_{c}^{T}E^{T}y_{k})\right]}^{\frac{N}{2}}\right\}\right]\underset{c}{\prod }\frac{1}{\left|\pi_{c}\right|}\mathrm{Pr}(E)\text{.}$$

Thus the voxelwise parameters do not need to be included within a numerical optimization for the parameter values, substantially reducing the computational complexity. If the encoding matrix and the class proportions are known (or estimated using Eq. (11)) then the flow can be calculated:

(12) $$f_{k}=E(f{\;}_{k}|y_{k})=\underset{c}{\sum }\mathrm{Pr}(q_{k}=c|y_{k}\text{,}E\text{,}\pi_{c})P_{c}{\left(P_{c}^{T}E^{T}{\mathrm{EP}}_{c}\right)}^{-1}P_{c}^{T}E^{T}y_{k}$$

where the class posterior probabilities are calculated from:

(13) $$\mathrm{Pr}(q_{k}=c|y_{k}\text{,}E\text{,}\pi_{c})=\frac{p_{k\text{,}c}}{\sum_{c}p_{k\text{,}c}}$$

where p_k,c_ is the non-normalized class label posterior probability:

(14) $$p_{k\text{,}c}=\pi_{c}[{\left(y_{k}-{\mathrm{EP}}_{c}{\left(P_{c}^{T}E^{T}{\mathrm{EP}}_{c}\right)}^{-1}P_{c}^{T}E^{T}y_{k}\right)}^{T}{\left(y_{k}-{\mathrm{EP}}_{c}{\left(P_{c}E^{T}{\mathrm{EP}}_{c}\right)}^{-1}P_{c}^{T}E^{T}y_{k})\right]}^{\frac{N}{2}}\text{.}$$

### 2.4 Encoding matrix entries

The encoding matrix, in Eq. (2), represents the combination of the applied encoding profile and the locations of the arteries within the labelling plane. In a general artery encoding scheme a number of encoded images will be acquired. Hence there will be a modulation value, *m*, for each combination of artery and modulation phase, i.e. for the *i*th encoded image and *j*th vessel:

(15) $$m_{i\text{,}j}=g(x_{j}\text{,}v_{j}\text{,}c_{i}\text{,}\theta_{i}\text{,}D_{i})\text{,}$$

where *g* is the 1-dimensional modulation function, ***x**_j_* is the location and v_j_ is the speed of flow in the jth artery, *c_i_*, *θ_i_* and *D_i_* are the centre, direction and scale of the *i*th encoding. The modulation function can be simulated by numerical evaluation of the Bloch equations for a range of mean flow speeds assuming a parabolic flow profile (Chappell et al., 2010; Wong, 2007). The modulation function is periodic and can be parameterised in terms of a non-dimensional phase *φ*, which can be evaluated for any combination of artery location and encoding setup from *φ = πd/*2*D*, where:

(16) $$d=\left\{\begin{array}{cc} \sin(x-c_{x})\sqrt{{\left(x-c_{x}\right)}^{2}+{\left(y-c_{y}\right)}^{2}}\cos\left(\theta -\arctan\left(\frac{y-c_{y}}{x-c_{x}}\right)\right) & x-c_{x}\;\neq \;0\\ (y-c_{y})\sin\theta & x-c_{x}=0 \end{array}\right)$$

In theory the vessel locations can be determined *a priori* from an angiographic image taken at the planning stage of the acquisition. However, there may be subsequent motion that results in a shift of the arteries from their expected location. In the next section we will consider two different approaches to overcome this problem. In one approach, the vessel locations can be added to the analysis as explicit further parameters to be determined from the data. This is the approach used in Chappell et al. (2010). In this case priors on the vessel locations are based on the expected locations from the planning angiographic image. However, the result of patient motion should be constrained to be a rigid body transformation of the artery locations. So we propose a new approach in which, instead of directly inferring artery locations, we infer parameters of a rigid body transformation, reducing the number of degrees-of-freedom in the analysis.

## 3 Methods

### 3.1 Analysis

A selection of analyses were considered to examine the trade-off in accuracy and robustness offered by the use of differing constraints in the analysis of VE-ASL angiographic data:

• Standard matrix inversion (**MI**) where the artery locations from a planning angiographic acquisition are used to form the encoding matrix according to Eqs. (15) and (16), assuming flow speed was 30 cm/s in each artery. The encoding matrix was (pseudo) inverted and used to calculate voxelwise flow contributions. In the case of simulated data, MI using the true artery locations and flow speeds was also calculated (**MIt**)
• ‘Bayesian inversion’ (**BI**): Bayesian analysis using fixed global parameters. Estimation of flow contributions using Eq. (12) assuming equal class proportions and using the artery locations from the planning acquisition to define the encoding matrix according to Eqs. (15) and (16) with flow speed 30 cm/s in each artery. This avoids the computationally costly estimation of ‘global’ parameters using Eq. (11) altogether.
• Bayesian inference of artery locations (**Bxy**): MAP solution of Eq. (11) with the encoding matrix parameterised by the locations of the arteries in the encoding plane, according to Eqs. (15) and (16). A prior was placed on the artery locations:

(17) $$\mathrm{Pr}{\left([x_{j}\;y_{j}]\right)}^{T}∼N(x_{j}\text{;}x_{0j}\text{,}1)N(y_{j}\text{;}y_{0j}\text{,}1)$$

where *x_0j_* and *y_0j_* are the estimates for the *j*th artery from the planning angiographic acquisition in mm. Estimation of flow contributions was then carried out using Eq. (12). In the case of simulated data, flow contributions using the MCMC solution of Eq. (9) was also calculated (**Bxy_MCMC_**) for comparison.

• Bayesian inference of rigid body transformation of artery locations assuming a 3 DOF transformation (**BT3**). This represents a simple model of patient movement after planning. The transformation can be described by a rotation, *θ*, and a 2D translation [*x_d_* *y_d_*]^T^:

(18) $$\left[\begin{array}{c} x_{j}\\ y_{j} \end{array}\right]=\left[\begin{array}{cc} \cos\;\theta & \sin\;\theta \\ -\sin\;\theta & \cos\;\theta \end{array}\right]\left[\begin{array}{c} x_{0j}\\ y_{0j} \end{array}\right]+\left[\begin{array}{c} x_{d}\\ y_{d} \end{array}\right]$$

where the transformation was performed about the centre of the artery locations. Informative priors were applied to the transformation parameters:

(19) $$\begin{array}{cc} & \mathrm{Pr}({\left[x_{d}\;y_{d}\right]}^{T})∼N(x_{d}\text{;}0\text{,}1{\mathrm{mm}}^{2})N(y_{d}\text{;}0\text{,}1{\mathrm{mm}}^{2})\\ & \mathrm{Pr}(\theta )∼N(0\text{,}{\left(5{}^{\circ}\right)}^{2}) \end{array}$$

Transformation parameters and class proportions were inferred using a MAP procedure via Eq. (11), and estimation of flow contributions was then carried out using Eq. (12).

• Bayesian inference of rigid body transformation and artery flow speeds (**BT3v**): This is the same as BT3 but with the artery flow speeds, in Eq. (15), being inferred as extra variables with prior:

(20) $$\mathrm{Pr}(v_{j})∼N(30\;\text{cm}/\text{s,}{\left(10\;\text{cm}/s\right)}^{2})$$

• Bayesian inference of rigid body transformation assuming a 6 DOF 2-dimensional affine transformation (**BT6**). Similar to BT3 but with the entries in the rotation matrix being inferred:

(21) $$\left[\begin{array}{c} x_{j}\\ y_{j} \end{array}\right]=\left[\begin{array}{cc} a & b\\ c & d \end{array}\right]\left[\begin{array}{c} x_{0j}\\ y_{0j} \end{array}\right]+\left[\begin{array}{c} x_{d}\\ y_{d} \end{array}\right]$$

with priors on the rotation matrix entries:

(22) Pr(a)∼N(1,0.1),Pr(b)∼N(0,0.1),Pr(c)∼N(0,0.1),Pr(d)∼N(1,0.1)

This extends the BT3 solution to a higher number of degrees-of-freedom to account for non-planar subject motion between planning and VE-ASL acquisition.

Analysis was performed in Matlab (MathWorks, Natick, MA) using code written in-house and was executed as a complied run time on an Intel Xeon Quad core server running at 3.0 GHz (all code was single threaded). MAP estimation of ‘global’ parameters was made using the BFGS Quasi-Newton method (Broyden, 1970; Fletcher, 1970; Goldfarb, 1970; Shanno, 1970) with a cubic line search (fminunc using the ‘Medium scale’ algorithm). To aid convergence of the inference to the global solution a multi-step evaluation was performed. Firstly vessel locations or transformation parameters were inferred with the class proportions fixed. Subsequently the class proportions were inferred with other parameters fixed, before a final stage where both class proportions and vessel locations or transformation parameters were inferred. For BT3v a further step was added at the end where the artery flow speeds were also inferred.

### 3.2 Simulations

**Fig. 2:**
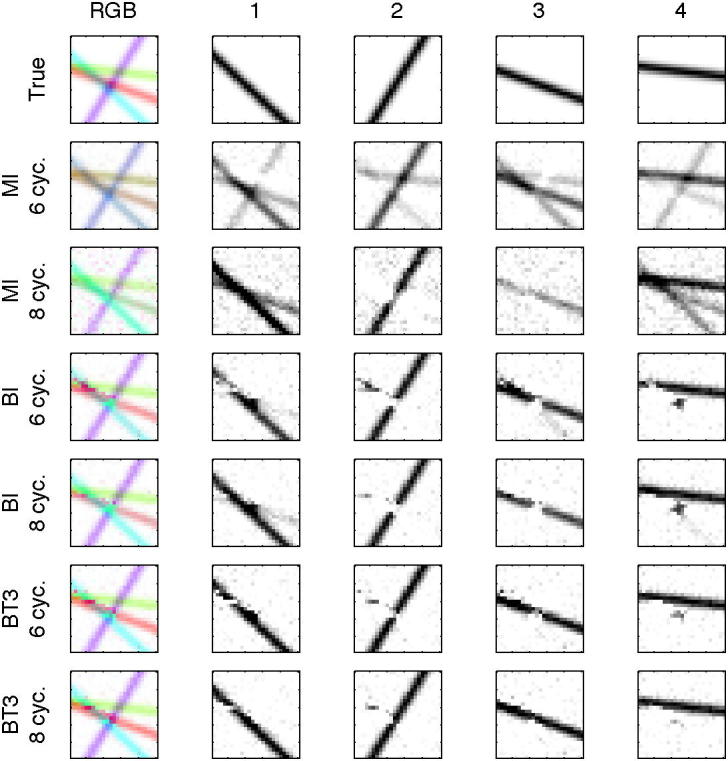
Example flow images from the analysis of one set of simulated data. Top row shows the true flow contributions from the four arteries within the imaging region used to generate the data. Results are shown from data generated with six or eight encoding cycles for analysis using matrix inversion (MI), ‘Bayesian inversion’ (BI) and Bayesian inference of a 3 DOF transformation (BT3). The left-most column shows the combined contributions from all four arteries using the RGB colour space (artery 1 = magenta, 2 = purple, 3 = red, 4 = green), the right four columns show the individual artery contributions. (For interpretation of the references to colour in this figure legend, the reader is referred to the web version of this article.)

To test the relative accuracy of the different analysis methods simulated data were generated that approximated a VE-ASL angiographic acquisition. The simulation included 4 arteries in the labelling plane placed at the corners of a square (±10 mm in *x* and *y*). These were treated as the locations obtained from the planning acquisition. For data generation these were subject to a two dimensional 3 DOF rigid body transformation with two translations and one rotation drawn randomly from a zero mean normal distribution with a standard deviation of 1 mm for translation and 1° for rotation. Additionally the flow speed in each artery was drawn from a normal distribution with mean 30 cm/s and standard deviation 5 cm/s (hard thresholds were applied for flow speeds exceeding 80 cm/s or falling below 3 cm/s). An imaging region 25 × 25 × 1 voxels was populated with four arterial segments whose centre and orientation was randomly generated, an example is shown in Fig. 2. Data were generated from eight encoding cycles detailed in Table 1, these represent a tag all, control all, two left–right, two anterior-posterior and two oblique encodings. The first six encoding cycles match the experimental design used in the real data (below), the final two oblique encoding cycles produce data for which the encoding matrix is full rank and thus matrix inversion analysis is not underdetermined. The parameters of the encoding cycles were based on the planning locations of the vessels prior to rigid body transformation to mimic a real experiment. One hundred datasets were generated with both 6 and 8 cycles with each measurement being subject to white noise with an SNR of 10:1 relative to the signal magnitude of the labelled blood. Data were analysed according to the methods outlined above. The root-mean-square-error (RMSE) between the estimated flow contributions from each artery and the true values was calculated. Since it is the detection of vessels in the brain and discrimination of the contributions from the labelled arteries that is typically of clinical interest, receiver operating characteristics (ROC) curves were also generated from the simulated data. This was achieved by taking the estimated flow contribution images and using a threshold to create a binary image representing the presence or absence of artery segments. Over a range of threshold values rates of true and false flow identification were used to calculate the ROC curves.

### 3.3 Real data

Two sets of VE-ASL dynamic angiographic data were considered to illustrate the proposed methods. Data had been acquired at 3 T using a Siemens TIM Trio system (Siemens Healthcare Erlangen, Germany) under a technical development protocol agreed with local ethics and institutional committees. The first subject was a young healthy volunteer with no known neurologic deficit. The second subject was a patient with severe bilateral vertebral artery stenoses scanned according to a protocol approved by the local ethics review board.

Prior to VE-ASL acquisition a standard time-of-flight (TOF) image was acquired of the head and neck. This image was used to estimate the locations of the arteries within the labelling plane and set up the modulations used within the VE-ASL acquisition itself. Acquisition was performed using the vessel-encoded pseudo-continuous ASL method of Wong (Wong, 2007), followed closely by a two-dimensional thick-slab dynamic angiographic readout, as described in (Okell et al., 2010). Labelling was achieved by a pulse train of 1 s duration using Gaussian pulses with an effective flip angle of 20°, 600 μs duration and 960 μs separation. 20 time points were acquired with a temporal resolution of 55 ms, allowing visualisation of the dynamics of the blood flow, using a segmented look-locker sampling strategy (Günther et al., 2001) with an excitation flip angle of 10°, 20 readout blocks with 3 phase encoding steps per readout block. Other readout parameters were: field-of-view 205 × 154 mm, matrix size 192 × 144, slab thickness 5 cm, total imaging time 10 min. The relatively long labelling duration used in this method means that the major cerebral vessels are filled with labelled blood at the first time point in the data, with subsequent time points showing outflow of the blood. Labelling was performed approximately 8 cm below the Circle of Willis where the two internal carotid and two vertebral arteries run approximately perpendicular to the transverse plane. Six VE-ASL cycles were performed: tag all, control all, tag left arteries whilst controlling right; tag right arteries whilst controlling left; tag anterior arteries (internal carotids) whilst controlling posterior; and tag posterior arteries (vertebrals) whilst controlling anterior.

Analysis was performed using the methods outlined above with the estimated artery locations from the TOF acquisition. The full complex magnitude and phase of the data was used for analysis, since substantial changes in the phase of signal from large vessels are possible, leading to artefacts in magnitude only data. For the methods where ‘global’ parameter estimation was required, the estimation was carried out on only the first time point image from within the dataset. This considerably reduced the size of the data to be processed and thus the computational cost. We used the first frame since it would contain the largest signal, as the ASL label still fills the major arteries at this stage. Flow parameter estimation was carried out using the full 20 time point data. The computation time for the parameter inference phase for each of the methods was recorded to give an indication of the relative speed of the different approaches.

## 4 Results

### 4.1 Simulated data

Fig. 2 shows RGB and individual artery contribution images from one set of simulated data. This set was chosen since it contained an area of overlap of multiple arteries in the imaging plane. Estimated images are shown from both six and eight cycle data for MI, BI and BT3 analyses with two arteries per class (APC), the other Bayesian methods produced images that were not visually distinguishable from BT3 in this case. The rank deficiency of the six cycle data was noticeable with MI, where there was substantial mixing between the different flow images. This was markedly reduced for the 8 cycle data. However, some mixing was still visible, a result of errors due to the movement of the vessel locations from their positions assumed for the calculation of the encoding matrix. Separation was good for all the Bayesian methods, the main source of separation errors occurring where three or more arteries overlapped.

**Fig. 3:**
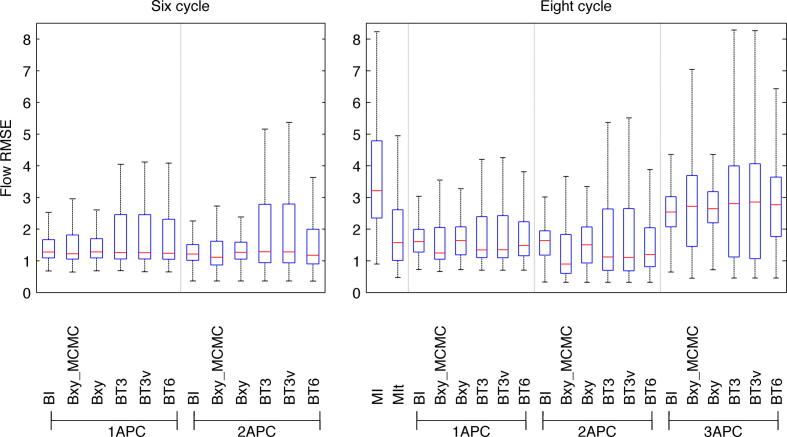
Root-mean-squared error (RMSE) between flow images and true flow contributions across the simulated datasets for the various analysis methods considered for both six and eight cycles of vessel-encoding. MIt for six cycles is not shown on these axes due to it having a median RMSE of 11.1.

**Fig. 4:**
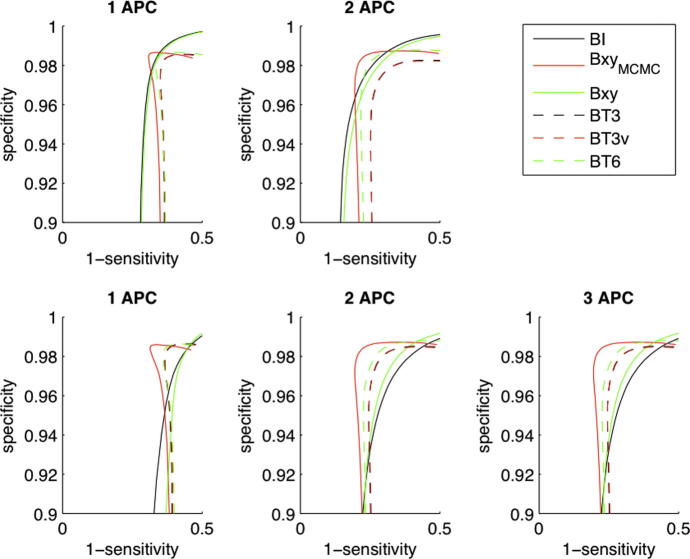
ROC curves for the identification of arteries from the simulated data: 6 cycle (upper) and 8 cycle (lower) data.

Fig. 3 shows the RMSE and Fig. 4 the ROC curves for the estimated flow contributions across all the simulated datasets for the analysis methods considered. Median error across the different datasets was smallest using two arteries per class (APC), although the difference was marginal for the data containing six cycles. Smaller errors were observed when the data contained the full eight cycles specified in Table 1. There was negligible difference in the error between the BT3 and BT3v cases and these offered a small improvement over the BT6 and Bxy alternatives. The smallest error and greatest discrimination was achieved using the MCMC rather than MAP solution (Bxy_MCMC_), although the improvement over a MAP solution was relatively small. Bayesian inversion (BI), while resulting in larger errors than the ‘global’ parameter search methods, still offered a marked improvement over standard matrix inversion analysis. Even when matrix inversion was performed using the true artery locations (MIt) the Bayesian inversion (using the ‘ideal’ locations) was comparable if not better.

### 4.2 Healthy subject data

**Fig. 5:**
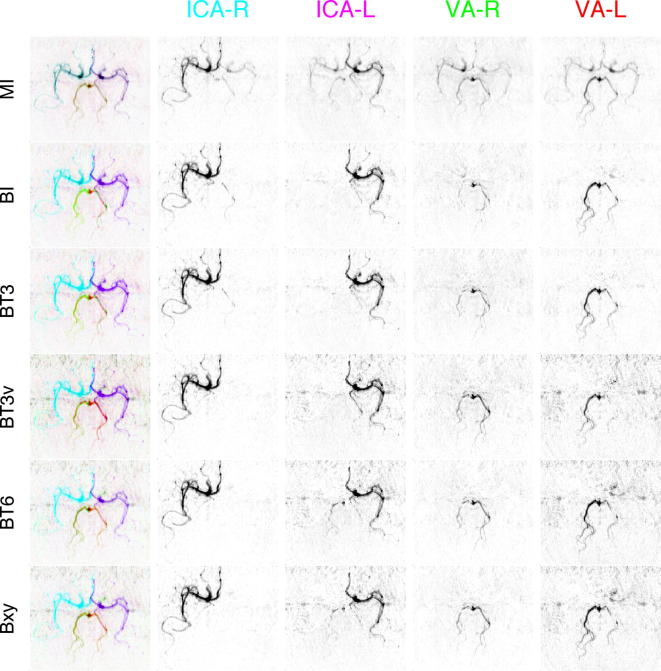
RGB flow image from a healthy subject: only the first frame from the dynamic angiographic acquisition of the Circle of Willis in the transverse view is shown. Combined (left-most) and individual artery contributions from internal carotid arteries (ICA) and vertebral arteries (VA) are shown. All the Bayesian analysis methods showed a broadly similar flow image, but with some variation in the posterior artery segments.

Fig. 5 shows the first frame from the healthy subject dataset analysed using the general framework with 2 APC. Video 1 gives the full dynamic sequence resulting from the BT3v analysis. The matrix inversion solution produced poor separation due to the rank deficiency of the encoding matrix, the dominant artery in a vascular segment was generally correct, but artefactual contributions from all other arteries were seen almost everywhere. The five different Bayesian analyses in Fig. 5 were broadly similar, although some differences can be seen in the posterior cerebral arteries. In general, the posterior cerebral artery territories are fed by the two vertebral arteries that fuse (in this case below the imaging region) to form the basilar artery, in which the vertebral blood may mix. It was apparent from all the methods that this mixed vertebral supply was present in the right posterior cerebral artery (PCA). It also appeared that the left PCA was being supplied by a combination of the mixed vertebral supply and flow from the left internal carotid artery (ICA) via the left posterior communicating artery (PCoA). Some contribution to the right PCA from the left ICA was seen for the BT6 and Bxy methods, this was considered likely to be an artefact since there was unlikely to be enough pressure to force blood from the left PCoA back along the proximal left PCA into the right PCA. Table 2 gives the computation time recorded for estimation of the ‘global’ parameters on this dataset.

### 4.3 Patient data

**Fig. 6:**
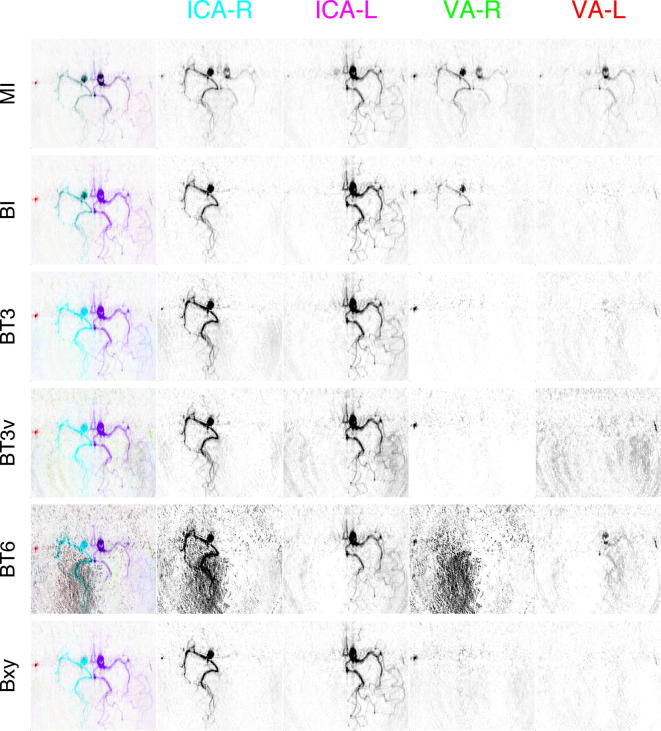
RGB flow images from a patient showing no flow contributions from the vertebral arteries. Combined (left-most) and individual artery contributions from internal carotid arteries (ICA) and vertebral arteries (VA) are shown. Effects of patient motion seen in the BI analysis are removed in the other methods; some artefactual appearance of the background noise can be seen in the BT6 results.

Fig. 6 shows the first frame from the patient dataset analysed using the general framework. In this case there was no apparent contribution from either vertebral, consistent with their clinical assessment. The posterior territories were being supplied via collateral flow from the ICAs through the PCoAs. This was evident from all the analysis methods based on the general framework, but as in the healthy subject, the separation was poor with MI. The BI method showed some contribution to the right cerebral arteries from the right vertebral. This appeared to be artefactual, primarily because the right vertebral ‘contribution’ very closely matched that of the right internal carotid. This artefact would be consistent with movement of the patient between planning and acquisition and the BT3 method reported a shift in the artery locations about their isocentre of 1.5 mm right, 3.2 mm anterior with 2.2° of rotation. The BT6 solution exhibited an increased contribution from background noise that was spread between the right carotid and vertebral components. In all the images a component from the right vertebral (red) could be seen on the right of the patient, this was identified as a branch of the right external carotid. It is likely that the right external carotid was labelled in a similar manner to the right vertebral due to its location in labelling plane.

## 5 Discussion and conclusions

In this work we have applied the general framework for VE-ASL analysis to dynamic angiographic images. This permits non-invasive visualisation of arteries in the brain allowing each artery’s signal contribution to be separately determined. To avoid the computationally costly MCMC evaluation of ‘global’ parameters in the original solution for VE-ASL perfusion imaging we have adopted MAP estimation. This coupled with only evaluating these parameters on a single frame of the dynamic sequence, leads to more acceptable processing time for high-resolution angiographic data. Typically processing times when performing MAP estimation of ‘global’ parameters up to 600 s were observed for the real data considered. For comparison a similar MCMC analysis would take of the order of 40,000 s on the same hardware. The actual time taken will vary in practice from dataset to dataset because an iterative optimization procedure is involved. An alternative that we have explored is calculating ‘global’ parameters within a mask defined from the tag-control difference signal, including only voxels containing substantial blood flow, across all time frames. Since this typically contains fewer voxels processing time is faster and by using all time frames it would be less sensitive to late arriving blood as might occur in stenotic or occlusive vascular diseases.

To improve robustness in a clinical context we modified the original general framework, reducing the number of free parameters associated with the artery locations in the labelling plane. This was achieved by using the artery locations from the planning acquisition, and then modelling transformations that might be expected due to subject motion. In simulation and real data this was shown to confer some improvement in accuracy, although the overall effect was relatively small. However, in some clinical cases there may be an advantage to being able to more closely define the relative locations of the arteries as achieved using the transformations in the BT3 and BT6 solutions. For example, in the patient case there is complete occlusion of the vertebral arteries leading to no signal from these in the resulting images. There is a danger that the Bxy algorithm might shift the vertebral locations to the location of a peripheral vessel that has been unintentionally included in the labelling region. In such circumstances it becomes harder to determine unambiguously from the results that no signal arises from the missing vertebrals, since it may be simply that a stronger signal has been observed from the peripheral vessel. By fixing the relative locations of the arteries it is possible to interpret the zero signal from the vertebrals with greater confidence, but still allow for patient movement.

We have shown that it is possible to avoid MAP parameter estimation altogether by calculating flow images from Eq. (12) using the assumed artery locations and equal class proportions. In terms of assuming known artery locations, this approach is similar to the matrix inversion approach, which uses a pseudo inversion of the encoding matrix. However, the MAP approach, which we have termed ‘Bayesian inversion’, also exploits the advantages of the classification built into the general framework, resulting in more accurate flow images, particularly in the face of a rank deficient encoding matrix. The reliance on accurate vessel locations means that BI is not most suitable when there has been motion between planning and acquisition, as seen in the patient dataset. However, it still shows a remarkable degree of robustness to artery location inaccuracy as demonstrated by the simulated data. Given the modest increase in computational complexity over matrix pseudo inversion we would propose that BI is a natural replacement for MI analysis in cases where images must be produced rapidly following the acquisition.

In this work we have adopted a MAP estimation of the ‘global’ parameters to obtain results within a clinically acceptable time frame. A possible disadvantage of this over the MCMC solution we have taken previously is that information about the uncertainty in the ‘global’ parameters is lost. However, the uncertainty associated with the ‘global’ parameters in this application is not critical. What is required is a point estimate that can be used to recover flow images from individual arteries. Another potential disadvantage is that a MAP method is more liable to find a local rather than the global minimum when compared with MCMC approaches. We might expect the posterior to possess multiple local minima because of the cyclic nature of the modulation functions. It is feasible that there would thus be multiple solutions for the vessel locations that result in very similar flow images. In practice the relatively informative priors used should avert this danger. Additionally the ‘global’ parameter values are not of direct interest (they can be considered nuisance parameters): it is the flow images that are desired. Thus as long as the flow image is correct, whether the estimated vessel locations match the true values or a cyclic equivalent is not an issue.

In this work we have considered data that has been acquired with the aid of a planning angiographic image of the labelling region. This image being used to define the encoding cycles to achieve maximum labelling efficiency and allowing the artery locations to be estimated. It may be desirable to have a planning free acquisition, for example using randomly defined encoding cycles as proposed by Wong and Guo (2012). In this case the vessel locations would be unknown and so a Bxy analysis would most likely be called for.

## Figures and Tables

**Table 1:** Setup of the vessel-encoded cycles used to generate simulated data.

Cycle	(*c_x_*, *c_y_*)	*θ*°	*D*
1	*Tag all*		
2	*Control all*		
3	(0, 0)	0	10
4	(20, 0)	0	10
5	(0, 0)	270	10
6	(0, 20)	270	10
7	(20, 20)	135	4.47
8	(20, 20)	135	4.47

**Table 2:** Computation times for ‘global’ parameter estimation (s) for the various Bayesian methods on the healthy dataset.

	1APC	2APC
BT3	117.8	207.1
BT3v	520.2	545.7
BT6	143.6	373.1
Bxy	380.3	201.9
