# Supplementary figures and images for: A fast analysis method for non-invasive imaging of blood flow in individual cerebral arteries using vessel-encoded arterial spin labelling angiography
Source: Med Image Anal. 2012 May;16(4):831–9. doi: 10.1016/j.media.2011.12.004 (PMC3398734; doi:10.1016/j.media.2011.12.004)

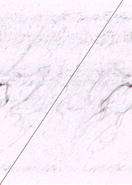

Supplement: Supplementary video 1 [file mmc1.jpg]

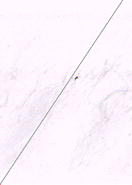

Supplement: Supplementary video 2 [file mmc2.jpg]
